# Diagnostic accuracy of blast-induced traumatic brain injury: a systematic review and meta-analysis

**DOI:** 10.3389/fneur.2026.1845916

**Published:** 2026-07-13

**Authors:** Peiqi Zhang, Lanxin Qin, Fulin Wang, Pengfei Wu, Liang Zhang, Danna Fang, Jingmei Zhao, Yuan Yao, Hui Zhao

**Affiliations:** 1 Department of Emergency, 903rd Hospital of Joint Logistic Support Force of PLA, Hangzhou, China; 2 Department of Military Traffic Injury Prevention and Control, Daping Hospital, Army Medical University, Chongqing, China

**Keywords:** blast injury, diagnosis, meta-analysis, systematic review, traumatic brain injury

## Abstract

**Objective:**

The purpose of this review was to systematically evaluate the existing diagnostic approaches of primary blast-induced brain injury through meta-analysis.

**Methods:**

A systematic review and meta-analysis were conducted in PubMed, Web of Science, Cochrane Library, and Defense Technical Information Center databases. Reference lists from the 15 identified studies mainly focused on clinical interviews, vestibular/ocular motor screening tools, neuroimaging, and non-specific biomarkers. Univariate meta-regression and subgroup analyses were employed to identify the sources of heterogeneity.

**Results:**

This meta-analysis demonstrated high diagnostic accuracy in included studies, the pooled sensitivity, specificity and AUC were 0.93 [95%CI: 0.81–0.97], 0.90 [95%CI: 0.80–0.95] and 0.96 [95%CI: 0.94–0.98], respectively. However, the choice of diagnostic tools should take clinical practice scenarios into consideration. Biomarker-based and neuroimaging approaches both achieved good diagnostic accuracy but were inaccessible in acute, resource-constrained battlefield scenarios. VOMS-based assessment may be more suitable for front-line and field use. Among structured clinical review tools, the BATL-2 instrument performed best in identifying primary blast-related TBI.

**Conclusion:**

Our findings demonstrate high diagnostic accuracy in the existing diagnostic methods for primary bTBI. However, each methods shows distinct applicability in different clinical scenarios. Much effort should be devoted to developing rapid, non-invasive and precise diagnostic approaches of primary bTBI in emergency settings.

**Systematic review registration:**

https://www.crd.york.ac.uk/PROSPERO/view/CRD420261281668; identifier: CRD420261281668.

## 1 Introduction

Primary blast-induced traumatic brain injury (bTBI), also termed blast-induced neurotrauma, refers to isolated primary blast injury to the brain caused by the initial overpressure and underpressure shock waves propagating through the tissue (1). It accounts for more than 20% of the incidence of TBI among Service members (SMs) on the battlefield (2). The primary blast wave is the most distinctive pathological feature of explosive trauma, differentiating bTBI from other TBIs caused by car accidents or sports (3). It can result in a wide spectrum of injury severity, ranging from mild concussion to severe TBI (3, 4). Inadequate triage protocols may delay or prolong diagnosis and treatment, leaving salvageable patients without timely life-saving interventions. The Defense and Veterans Brain Injury Center reported that roughly 500,000 U.S. military population were diagnosed with bTBI since 2,000 (5). However, bTBI is frequently underdiagnosed by clinicians, especially when comorbid with post-traumatic stress disorder and depression (6, 7). Given the high prevalence and insidious symptoms of bTBI, its impacts on SMs’ mission capabilities have attracted widespread research attention. Evidence shows that symptoms of bTBI could impair soldiers’ operational combat capacity (8–10). Common symptoms include cognitive dysfunction, memory loss, attention difficulties, visual alterations, vestibular disorders and impaired balance (11–13). SMs with mTBI may have difficulties in receiving orders and delayed reaction times, directly compromising battlefield readiness and post-deployment quality of life (14, 15).

To address the challenges in evaluating primary bTBI, a variety of measures have been proposed, including clinical interviews, vestibular and ocular screening tools, neuroimaging, and biomarkers. Semi-structured interviews are the mainstay for retrospective diagnosis of prior TBI. Representative tools include the Boston Assessment of TBI Lifetime (BAT-L) (16), the Ohio State University TBI Identification Method (OSU TBI-ID) (17), and the Virginia Commonwealth University Retrospective Concussion Diagnostic Interview (VCU rCDI) (18). These approaches are used alongside the Glasgow Coma Scale (GCS) for TBI severity grading: mild (13–15 points), moderate (9–12 points), and severe (<8 points) (19). Their assessments rely on patient-reported clinical signs and symptoms such as loss of consciousness, altered mental status and post-traumatic amnesia. Despite the widespread application, researchers continue to refine these tools through multiple strategies. For instance, unlike conventional TBI-focused scales such as BAT-L, the Salisbury Blast Interview (SBI) broadens its assessment to capture lifetime blast events. It also documents environmental conditions, protective gear use, and blast-related features, such as pressure and debris (20). Complementary assessments such as the Balance Error Scoring System (BESS) and Sensory Organization Test (SOT) strengthen diagnostic ability by evaluating postural instability (21). Clinical interview for bTBI continue to be an active field of research.

Another widely discussed approaches in the literature is the Vestibular/Ocular Motor Screening (VOMS). It consists of seven items: smooth pursuits, convergence, near-point convergence distance, horizontal and vertical saccades, horizontal and vertical vestibulo-ocular reflex, and visual motor sensitivity (22). Recently, it has been integrated into the Military Acute Concussion Evaluation 2 (MACE-2) assessment, a standard tool for acute concussion assessment (23). Computerized eye-tracking systems and machine learning models built on pupil measurement data have also become as viable diagnostic techniques in civilian settings. Anthony et al. developed a smartphone application to distinguish severe TBI patients from healthy controls. The tool adopted a machine learning algorithm using pupillometry data and achieved a good accuracy of 93.5% (24, 25).

Within neuroimaging techniques, CT remains the first-line imaging measures for acute TBI (26). However, it has low sensitivity for mild TBI (mTBI), with only 16.1% of mTBI patients exhibit abnormal findings (27). Diffusion tensor imaging (DTI) can detect cortical network disruptions caused by diffuse axonal injury, which underlie subsequent brain dysfunction (28, 29). Yet its discriminatory performance between mTBI and health controls is limited (30, 31). As an alternative, magnetoencephalography (MEG) emerges as a promising approach for detecting mTBI. It measures magnetic signals generated by neuronal activation in the gray matter (32). Biomarker-based approaches offer superior specificity and a distinct advantage in distinguishing cases with non-specific symptoms (33). Various biomarkers have been investigated: UCH-L1, GFAP, iNOS, S100B, C-tau, NSE, MBP, IL-8, IL-10, and various others (34–39). Among these biomarkers, S100B has been the most extensively studied biomarker in patients with mTBI (40, 41).

Despite the availability of diverse diagnostic approaches, comprehensive reviews summarizing diagnostic methods and their performance assessment of primary bTBI remain scarce. To address this research gap, this review aims to summarize existing diagnostic approaches (e.g., clinical review, screening tools, diagnostic algorithms and other emerging methods) for primary bTBI and conduct a meta-analysis to evaluate the predictive accuracy, providing empirical evidence for the upcoming related studies.

## 2 Methods

### 2.1 Study design and registration

The study design of this review followed the PICOTS framework (42). Participants included military personnel, breachers, explosive handlers, civilians, and laboratory animals, all of whom were either diagnosed with primary bTBI or served as controls. Index tests referred to all available diagnostic approaches for primary bTBI. Given the lack of a consensus reference standard for primary bTBI, we adopted liberal diagnostic criteria, including clinical diagnosis, structured interviews, neuroimaging and pathological examinations. These criteria varied across included studies. The outcomes were diagnostic metrics such as sensitivity and specificity. Index tests were performed across all post-injury stages: acute and chronic phases. No restriction was applied to study settings. This review was conducted in accordance with the Preferred Reporting Items for Systematic Reviews and Meta-Analyses (PRISMA) guidelines (43) and was registered in the PROSPERO platform on January 4th, 2026 (registration number: CRD420261281668).

### 2.2 Eligibility criteria

The inclusion criteria were as follows: (1) studies focused on primary bTBI resulting from blast exposure and repetitive blast-related neurological stresses (44); (2) cross-sectional, retrospective and prospective diagnostic studies were all eligible, with cross-sectional designs prioritized; (3) reported sufficient data to construct 2 × 2 contingency tables for calculating sensitivity and specificity; (4) eligible reference standards included clinical diagnosis, structured interviews, neuroimaging examinations, and pathological examinations; (5) no language restrictions were applied to literature retrieval. The exclusion criteria were as follows: (1) studies focusing on injuries caused by sports and motor vehicle collisions; (2) polytrauma; (3) *in vitro* or computer simulation models; (4) studies from which key indicators such as sensitivity and specificity could not be extracted; (5) case reports, systematic reviews and meta-analyses, conference abstracts; (6) prognostic studies; (7) publications without accessible full texts or complete raw data.

### 2.3 Search strategy

Two investigators independently conducted a literature search across the following databases: PubMed, Web of Science, Cochrane Library, and Defense Technical Information Center database. Search keywords were as follow: “blast,” “explosive,” “traumatic brain injury,” “TBI,” “accuracy,” “diagnostic,” “AUC,” “sensitivity,” “specificity.” Searches were conducted from inception to January, 2026. Any discrepancies were resolved via discussion with a third reviewer until a consensus was reached. Database search strategies and results are presented in Supplementary Table S1.

### 2.4 Study selection and data extraction

All retrieved studies were screened and organized using Rayyan software. Basic characteristics included the first author, year of publication, country, study design, sample size (total numbers as well as the number of bTBI cases and healthy controls), diagnostic method, age, and males proportion within bTBI cases, post-injury interval, sample type, diagnostic modality and reference. Classification of diagnostic modalities was based on the 2015 Institute of Medicine (IOM) report, *Improving Diagnosis in Health Care* (45), which divided diagnostic methods into clinical history and interview, physical exam, and diagnostic testing, including laboratory medicine, anatomic pathology and medical imaging. Accordingly, we reclassified diagnostic modalities into four groups: standardized interview and scales, physical exam, laboratory testing, and imaging with computational models. In terms of post-injury staging, we defined bTBI occurring within 72 h post-blast as acute and subsequent cases as chronic, as the initial 72-h is a critical window for acute pathological progression following blast trauma. For diagnostic performance analysis, true positive(TP), false negative(FN), false positive(FP), and true negative(TN) values were extracted to construct 2 × 2 contingency tables. When multiple models were reported in a study, the model with the highest diagnostic accuracy was selected for meta-analysis. Any discrepancies during screening and data extraction were resolved through consensus discussion.

### 2.5 Risk of bias and quality assessment

The methodological quality of all included studies was evaluated by the Quality Assessment of Diagnostic Accuracy Studies-2 (QUADAS-2) tool (2011 version) using Review Manager 5.4 software. QUADAS-2 consists of four domains: patient selection, index test, reference standard, and flow and timing (46). Two reviewers independently evaluated each domain and rated the risk of bias as low, unclear, or high for individual studies. Overall publication bias was assessed by Deeks’ test and funnel plots, with *p* < 0.05 denoting publication bias.

### 2.6 Statistical analysis

Meta-analyses were conducted in Stata MP 17.0 software, using the bivariate generalized linear mixed-effects model. Diagnostic accuracy was assessed using sensitivity, specificity, positive likelihood ratio, negative likelihood ratio, diagnostic odds ratio, and their corresponding 95% confidence intervals (CIs). Summary receiver operating characteristic (SROC) curves and areas under the curve (AUC) were utilized to evaluate the overall diagnostic performance. Heterogeneity was quantified using the *I*^2^ statistic and Cochran’s Q test. A fixed-effects model was used when *I*^2^ < 25%, while *I*^2^ ≥ 50% indicated high heterogeneity, and meta-regression and subgroup analyses were employed to investigate possible sources of heterogeneity. Publication bias was assessed using Deeks’ test and funnel plots, with *p* < 0.05 denoting publication bias. Diagnostic utility was further evaluated using Fagan nomograms and likelihood ratio scatter plots. Residual diagnostics, influence analysis, and outlier testing were utilized to verify fitting robustness of the meta-regression model. A bivariate scatter plot, chi-plot, and box plot were adopted for heterogeneity detection of included studies. *p* value < 0.05 was considered statistically significant.

## 3 Results

### 3.1 Search results

**Figure 1:**
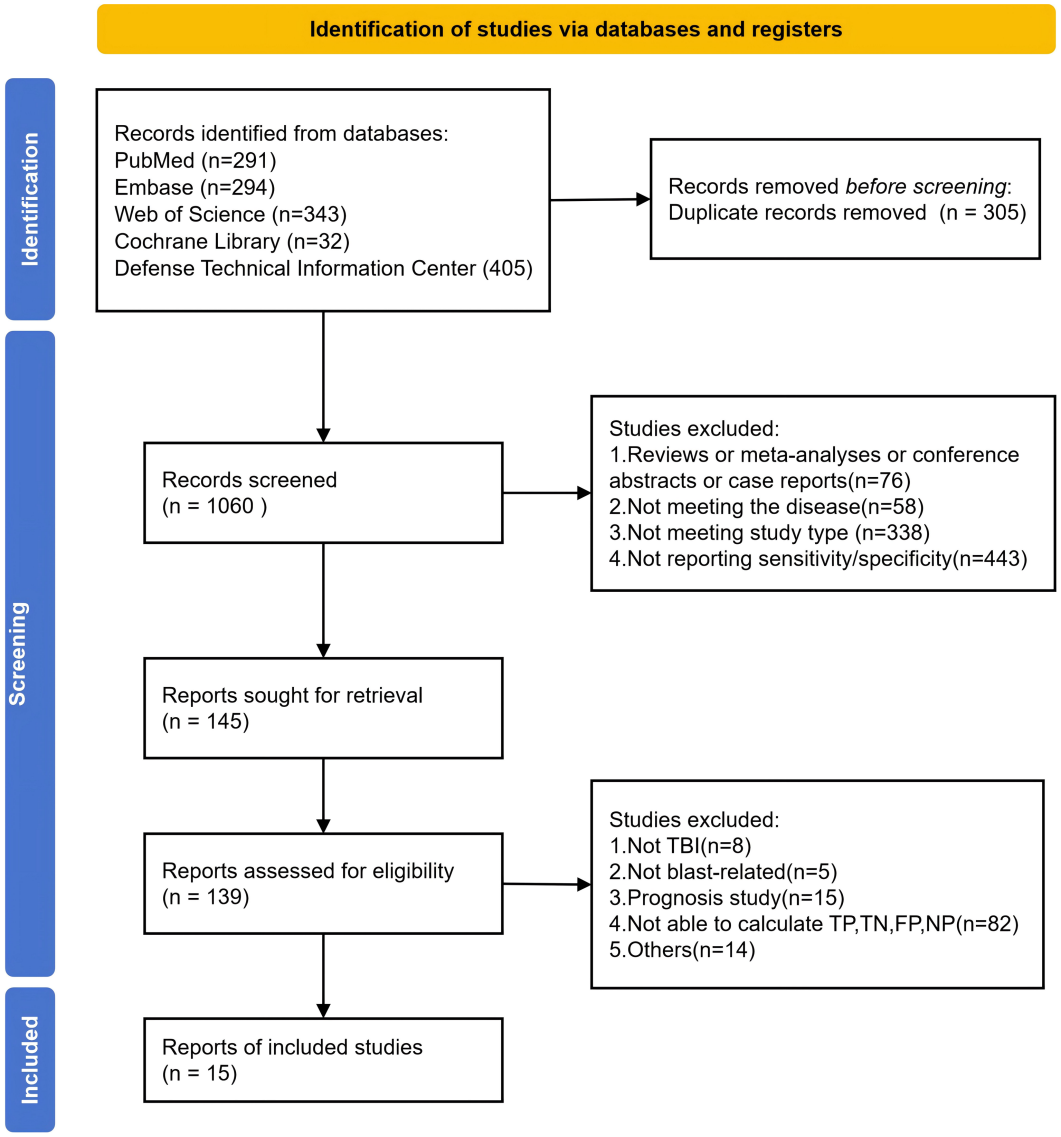
PRISMA flow chart.

Following the PRISMA guidelines, a total of 1,365 records were identified through databases: 293 from PubMed, 343 from Web of Science, 32 from Cochrane Library, and 405 from the Defense Technical Information Center. After removing 305 duplicate records, 1,060 irrelevant studies were excluded by reviewing titles and abstracts. Of the 139 studies assessed for full-text eligibility, 124 were excluded according to the inclusion and exclusion criteria, and finally, 15 studies were included. The study selection process was shown in Figure 1.

### 3.2 Basic characteristics of studies

The baseline characteristics of the 15 included studies are summarized in Table 1. Of these, 11 were conducted in human participants and four used animal models. The human studies enrolled a total of 2,377 participants, including 1,048 bTBI patients and 1,329 healthy controls. Four preclinical studies involved 204 mice, with 118 mTBI cases and 86 sham controls. In terms of study design, nine studies adopted a retrospective cross-sectional design, three applied prospective longitudinal study design, two employed a prospective cohort study design and one was a prospective cross-sectional design. Geographically, 11 studies were conducted in the United States, three in China, and one in Canada. The mean age of bTBI patients ranged from 24 to 43 years, and the proportion of male patients ranged from 86.2 to 100%. For blast-induced TBI diagnostic methods, five relied on standardized clinical interviews or diagnostic scales, four applied physical exams such as olfactory and visual screening, four utilized laboratory testing methods, and two studies used medical imaging data with computational models. Regarding the reference standard for TBI diagnosis, nine studies adopted comprehensive clinical diagnosis, one employed imaging diagnosis, one used structured interview, four animal studies utilized pathological examination. All studies provided sufficient information to calculate the numbers of FP, FN, TP and TN.

**Table 1:** Study characteristics.

Author	Year	Country	Study design	Diagnostic method	Human/Animal	Sample size	Age in bTBI group	% of Male in bTBI group
Andrea (55)	2025	USA	Retrospective cross-sectional	Logistic regression model with fMRI scans data	Human	424	43	99.50%
Anthony (23)	2024	USA	Retrospective cross-sectional	Vestibular/Ocular Motor Screening (VOMS)	Human	25	42.39	100.00%
Grant (56)	2019	USA	Retrospective cross-sectional	Automated Neuropsychological Assessment Metrics Version 4 TBI-MIL (ANAM4 TBI-MIL)	Human	733	26.9	100.00%
Huang (51)	2020	USA	Retrospective cross-sectional	3D-MEGNET model with rs-MEG source-magnitude imaging data	Human	101	29.86	100.00%
Jared (20)	2020	USA	Retrospective cross-sectional	The Salisbury Blast Interview (SBI)	Human	287	41.7	86.20%
Jose (57)	2012	USA	Retrospective cross-sectional	Computerized oculomotor vision screening (COVS)	Human	40	29.7	90.00%
Kranfli (52)	2025	USA	Prospective cohort	The Modified Vestibular/Ocular Motor Screening Tool (mVOMS)	Human	57	25	100.00%
Michael (58)	2015	USA	Prospective cohort	Olfactory impairment test	Human	231	25	96.60%
Sarah (59)	2023	USA	Retrospective cross-sectional	Military occupational specialty classification model	Human	256	41.56	86.30%
Tristan (53)	2025	USA	Retrospective cross-sectional	The Boston assessment of traumatic brain injury lifetime, second edition (BATL-2)	Human	120	34	91.70%
William (18)	2014	USA	Retrospective cross-sectional	The VCU retrospective concussion diagnostic interview, blast version (VCU rCDI-b)	Human	103	24	99.00%
Ge (60)	2023	China	Prospective longitudinal	PCA model with Raman spectroscopy data of hippocampus and hypothalamus tissues	Mice	45	/	100.00%
Ge (61)	2024	China	Prospective longitudinal	LDA model with Raman spectroscopy data of hippocampus and hypothalamus tissues	Mice	55	/	100.00%
Nam (33)	2014	Canada	Prospective cross-sectional	soluble cellular prion protein (PrPC)	Mice	52	/	100.00%
Wang (62)	2020	China	Prospective longitudinal	SVM algorithm with ATR THz-TDS data of serum and cerebrospinal fluid (CSF)	Mice	52	/	100.00%

Author	Sample type	Diagnostic modality	Reference	Post-injury interval	Phase	TP	FP	FN	TN
Andrea (55)	Brain imaging	Imaging with computational model	Clinical diagnosis	Not specified	/	187	70	25	142
Anthony (23)	Oculovestibular system	Physical exam	Clinical diagnosis	Within 60 days	Chronic	16	0	2	7
Grant (56)	Neuropsychology system	Standardized interview and scales	Clinical diagnosis	11 (0, 245) days	Chronic	42	237	14	440
Huang (51)	Brain imaging	Imaging with computational model	Clinical diagnosis	Not specified	/	59	0	0	42
Jared (20)	Neuropsychology system	Standardized interview and scales	Structured interview	>10 years	Chronic	206	29	24	28
Jose (57)	Oculovestibular system	Physical exam	Clinical diagnosis	1–10 years	Chronic	20	1	0	19
Kranfli (52)	Oculovestibular system	Physical exam	Clinical diagnosis	24 h post-injury	Acute	29	3	13	12
Michael (58)	Olfactory system	Physical exam	Imaging diagnosis	14.5 (0, 23) days	Chronic	62	0	114	55
Sarah (59)	Clinical classification	Standardized interview and scales	Clinical diagnosis	>10 years	Chronic	45	30	43	138
Tristan (53)	Neuropsychology system	Standardized interview and scales	Clinical diagnosis	Not specified	/	60	6	0	54
William (18)	Neuropsychology system	Standardized interview and scales	Clinical diagnosis	9.1 (6.3–9.7) months	Chronic	83	2	4	14
Ge (60)	Brain tissue	Laboratory testing	Pathological examination	3 h, 24 h, 48 h, 72 h	Acute	19	1	1	24
Ge (61)	Brain tissue	Laboratory testing	Pathological examination	3 h, 6 h, 24 h, 48 h, 72 h	Acute	24	1	1	29
Nam (33)	Body fluid	Laboratory testing	Pathological examination	24 h	Acute	26	4	7	15
Wang (62)	Body fluid	Laboratory testing	Pathological examination	3 h, 6 h, 24 h	Acute	40	1	0	11

### 3.3 Publication bias and quality assessment

**Figure 2:**
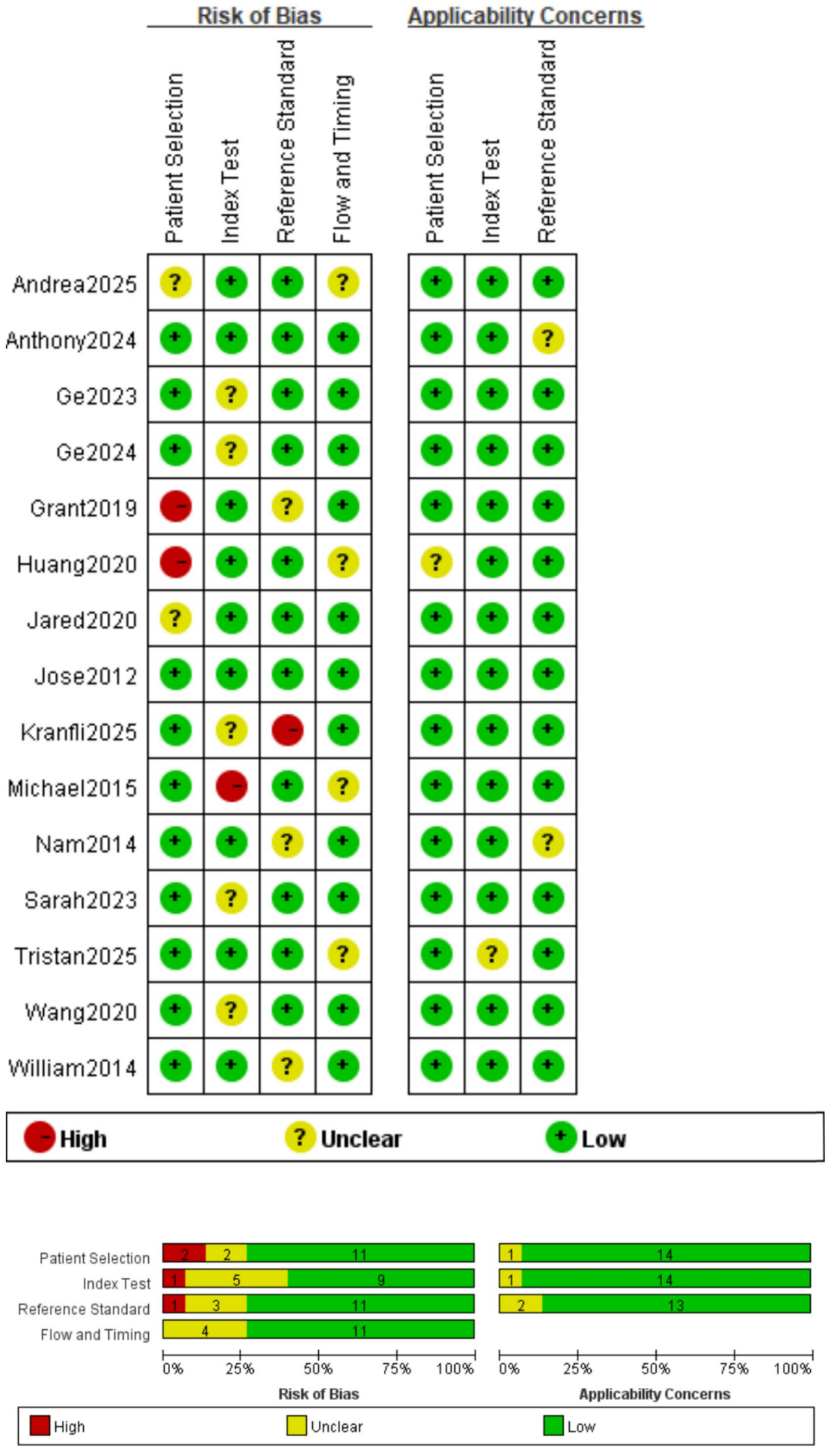
Quality assessment of included studies based on QUADAS-2 tool criteria.

**Figure 3:**
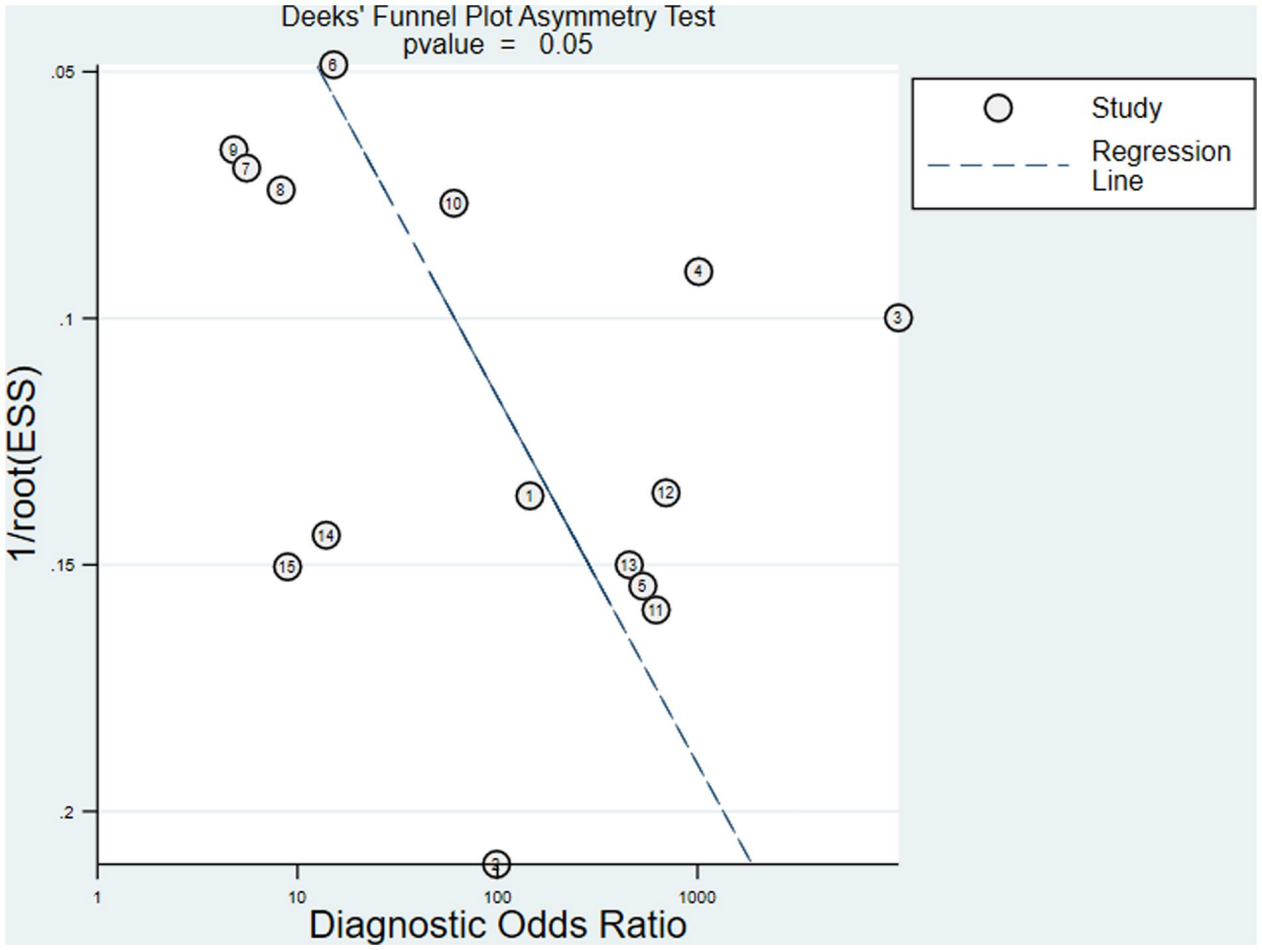
Deek’s funnel plot of the publication bias test of included studies.

According to the QUADAS-2 assessment results, most studies had a low or unclear risk of bias for the index test domain. High risk of bias was identified in two studies in the patient selection domain and one study in the index test and reference standard, respectively. For applicability concerns, only one study was rated as high concern in the reference standard domain. Details were presented in Figure 2. The Deeks’ funnel plot was symmetric (*p* = 0.054), indicating no statistically significant publication bias, as shown in Figure 3.

### 3.4 Diagnostic performance evaluation

**Figure 4:**
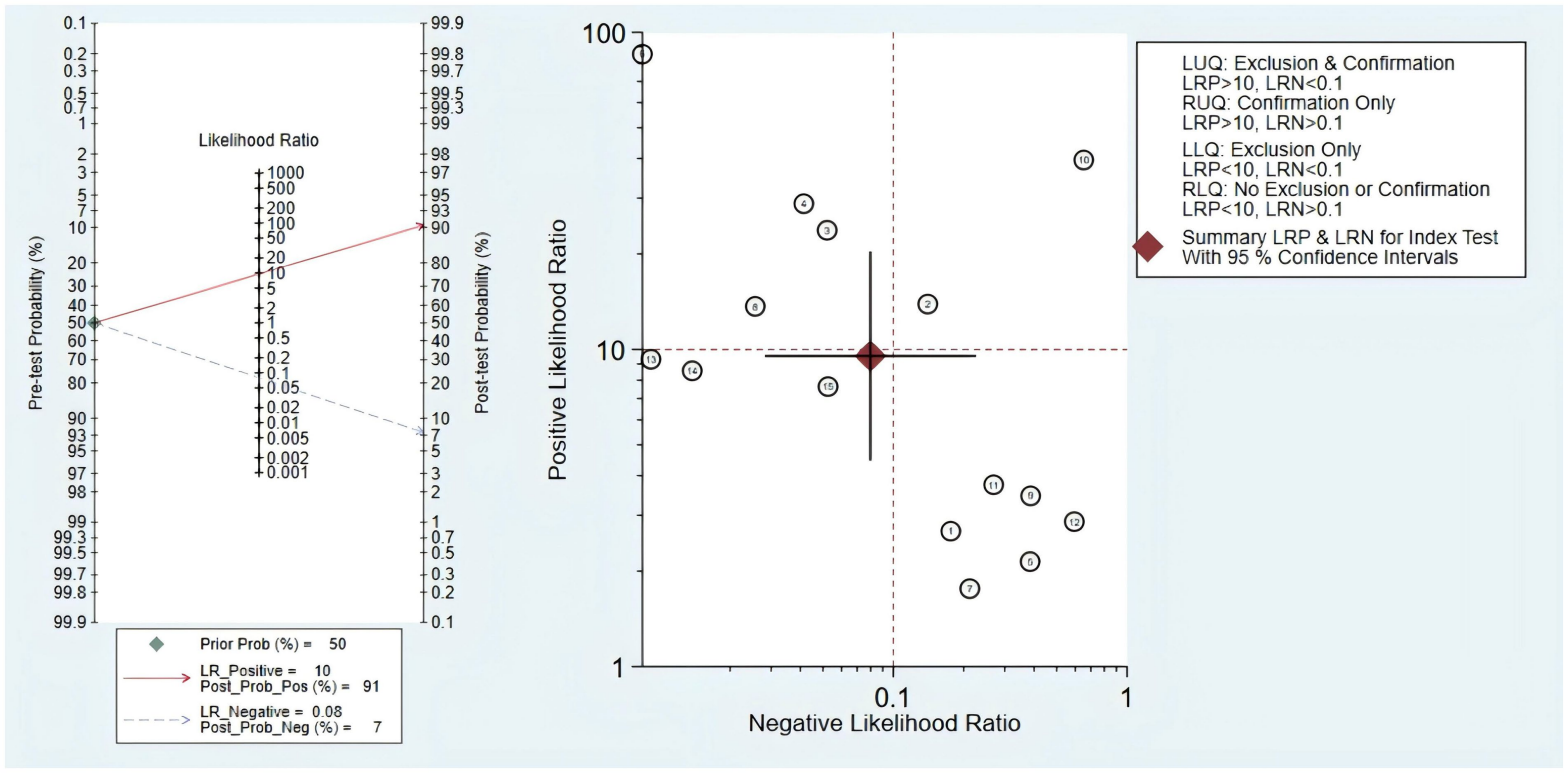
Fagan nomogram and likelihood ratio scatter plot. The left panel shows a Fagan nomogram. The right panel is a Fagan nomogram illustrating the post-test probabilities corresponding to positive and negative test results when the pre-test probability is 50%.

Figure 4 illustrated the diagnostic performance of included studies. The left panel showed a Fagan nomogram. At a pre-test probability of 50%, a positive test result increased the post-test probability to 91%, whereas a negative test result decreased the post-test probability to 8% (PLR = 10, NLR = 0.08, *p* < 0.001). The right panel depicted a scatter plot of positive and negative likelihood ratios of each included study. The pooled positive likelihood ratio was very close to 10, and the pooled negative likelihood ratio stood at 0.08, indicating that a positive result significantly increased the probability of bTBI, and a negative result effectively reduced the probability of disease. These results demonstrated that current diagnostic tools exhibited high overall accuracy for the evaluating primary bTBI. To verify the fitting performance of the meta-regression model, we also conducted model robustness tests, as shown in Supplementary Figure S1. Residual and influence analyses confirmed the normality and the absence of outliers, supporting the stability and reliability of the model.

### 3.5 Data synthesis result

**Figure 5:**
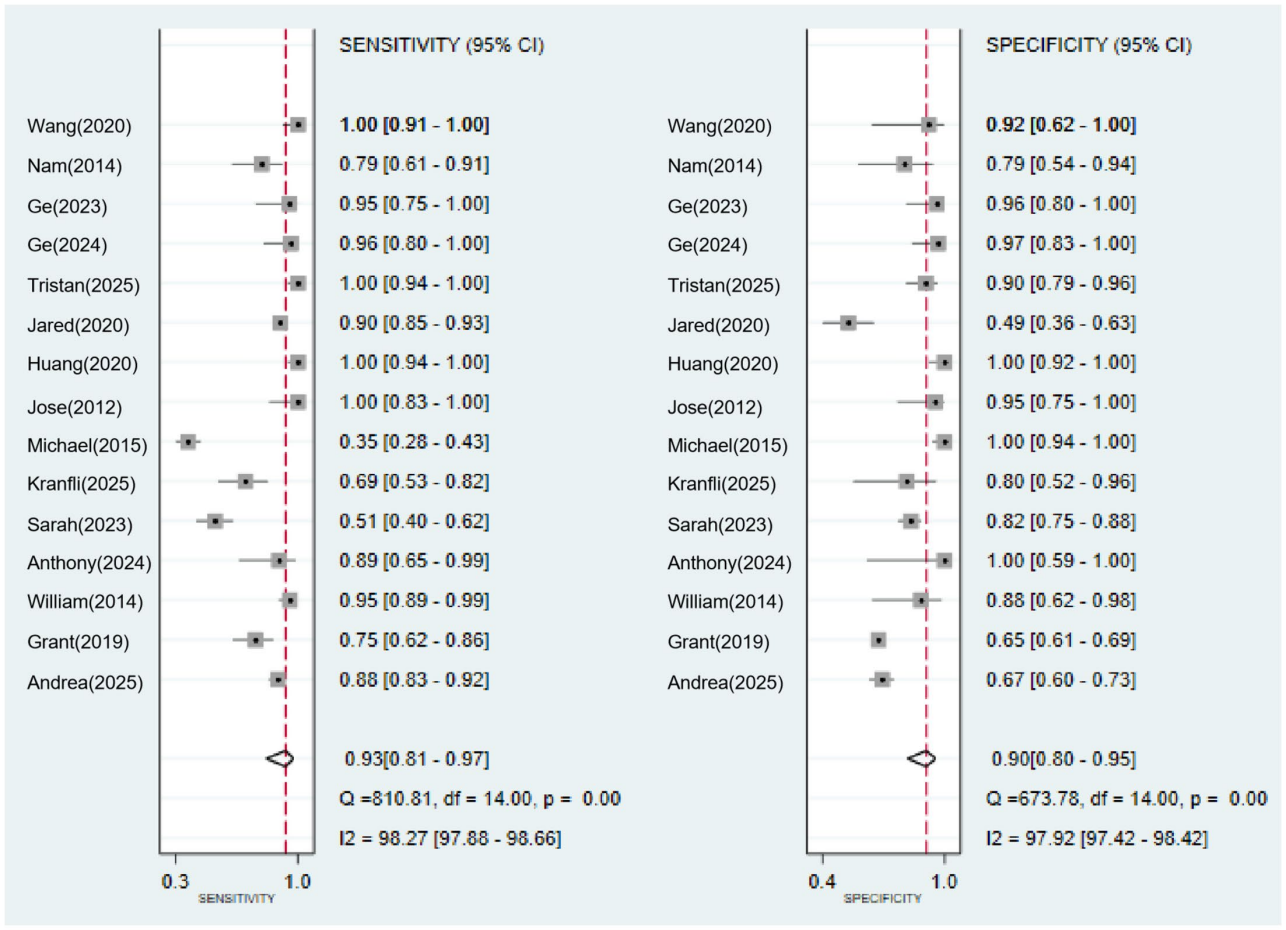
Forest plot of sensitivity and specificity with 95%CI of included studies.

**Figure 6:**
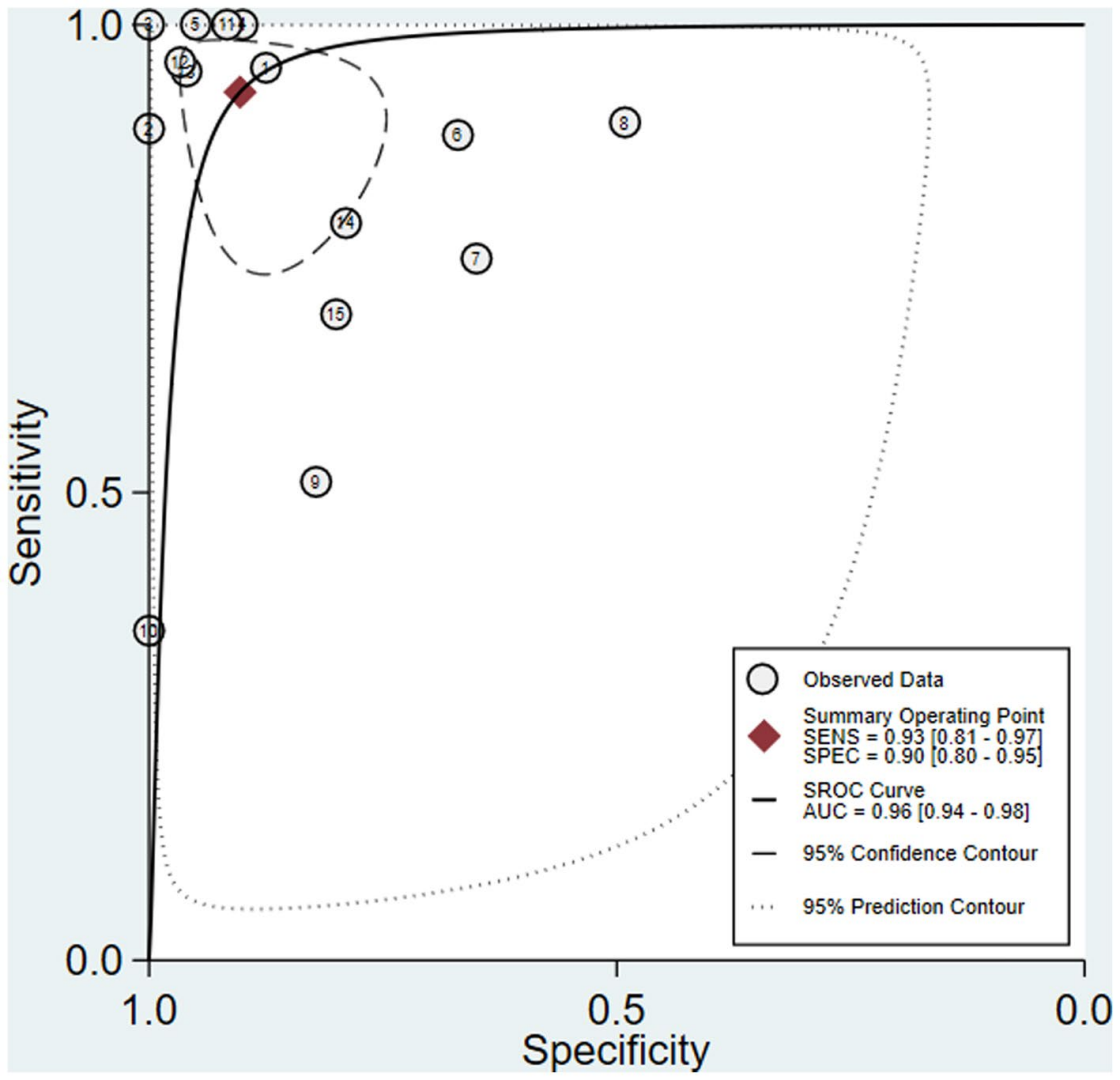
Summary receiver operating characteristic (SROC) curve of the included studies.

Pooled diagnostic performance across the included studies was presented in Table 2. Overall, the pooled sensitivity was 0.93 [95%CI: 0.81–0.97] and the pooled specificity was 0.90 [95%CI: 0.80–0.95]. The pooled positive likelihood ratio (PLR) was 9.5 [95%CI: 4.5–20.4], indicating a positive test increased the likelihood of bTBI more than nine times compared with the baseline. The pooled negative likelihood ratio (NLR) was 0.08 [95%CI: 0.03–0.23], well below 0.1, signifying that a negative result reduced the likelihood of bTBI to 8% of the pre-test probability. The pooled AUC was 0.96 [95%CI: 0.94–0.98], which confirmed high overall diagnostic accuracy. The relatively large 95% confidence and prediction ellipses on the SROC plot revealed substantial heterogeneity among included studies. Stratified by species, the 11 human studies yielded a pooled sensitivity and specificity of 0.91 [95%CI: 0.73–0.97] and 0.89 [95%CI: 0.75–0.96], respectively, with an AUC of 0.96 [95%CI: 0.94–0.97]. Four animal studies exhibited superior diagnostic efficacy, with sensitivity reaching 0.96 [95%CI: 0.82–0.99], specificity of 0.93 [95%CI: 0.81–0.98] and an AUC of 0.98 [95%CI: 0.96–0.99]. In general, the evaluated methods achieved high diagnostic performance for primary bTBI. The corresponding forest plot and SROC curve were depicted in Figures 5, 6, respectively.

**Table 2:** Pooled diagnostic performance.

Variables	All studies (*n* = 15)	Human (*n* = 11)	Mice (*n* = 4)
Positive samples, *n*	1,166	1,048	118
Negative samples, *n*	1,415	1,329	86
Sensitivity, 95%CI	0.93 (0.81–0.97)	0.91 (0.73–0.97)	0.96 (0.82–0.99)
Specificity, 95%CI	0.90 (0.80–0.95)	0.89 (0.75–0.96)	0.93 (0.81–0.98)
PLR, 95%CI	9.5 (4.5–20.4)	8.6 (3.3–22.5)	14.1 (4.6–42.7)
NLR, 95%CI	0.08 (0.03–0.23)	0.10 (0.03–0.34)	0.05 (0.01–0.22)
DOR, 95%CI	120 (26–543)	86 (14–523)	292 (29–2,962)
AUC, 95%CI	0.96 (0.94–0.98)	0.96 (0.94–0.97)	0.98 (0.96–0.99)

### 3.6 Heterogeneity analysis

In heterogeneity analysis, the *I*^2^ values for the pooled sensitivity and specificity across all included studies were 98.91% (95%CI: 98.87–99.15%, Cochran Q test *p* < 0.001) and 98.27% (95%CI: 95.05–97.49%, Cochran Q test *p* < 0.001), respectively, indicating substantial heterogeneity among included studies. We further calculated the *I*^2^ and Q value in human and animal studies separately. Significant heterogeneity was mainly observed in human studies (*I*^2^ = 99.95%, *p* < 0.001), whereas no heterogeneity was found in animal studies (*I*^2^ = 0%, *p* = 0.404). For human-related studies, the pooled sensitivity and specificity were 0.91 [95%CI: 0.73–0.97] and 0.89 [95%CI: 0.75–0.96], respectively, lower than those of animal-related studies (sensitivity: 0.96 [95%CI: 0.82–0.99], specificity: 0.93 [95%CI: 0.81–0.98]). This suggested that mouse model studies exhibited better diagnostic performance, which might be due to controlled experiment conditions and homogeneous samples.

### 3.7 Subgroup analysis

Univariate meta-regression and subgroup analyses were conducted among human studies. As presented in Table 3, Diagnostic modality were marginally associated with between-study heterogeneity (*p* = 0.05, *I*^2^ = 67%). No significant subgroup differences were observed for age, reference type and bTBI phase (*p* > 0.05). Results showed that physical exams such as VOMs exhibited the highest pooled specificity (0.97, [95%CI: 0.92–1.00]), while standard interview and scales presented relatively higher sensitivity (0.90, [95%CI: 0.77–1.00]). Neuralimaging with algorithm approaches achieved excellent overall diagnostic performance, with a sensitivity of 0.96 [95%CI: 0.85–1.00] and a specificity of 0.89 [95%CI: 0.69–1.00]. We further performed a bivariate analysis to evaluate sources of between-study heterogeneity. As shown in Supplementary Figures S2, S3, most points were in the 95%CI area in Scatter plot and bivariate boxplot, confirming the reliability of the diagnostic model.

**Table 3:** Univariate regression and subgroup analysis in human studies.

Variables	Chi square	*I*^2^ (%)	*p* value	Number of studies	Sensitivity (95%CI)	Specificity (95%CI)
Age	1.97	0	0.37	11	0.90 [0.74–0.96]	0.87 [0.75–0.94]
Diagnostic modality	5.98	67	0.05			
Physical exam				4	0.81 [0.55–1.00]	0.97 [0.92–1.00]
Imaging with algorithm				2	0.96 [0.85–1.00]	0.89 [0.69–1.00]
Standardized interview and scales				5	0.90 [0.77–1.00]	0.77 [0.59–0.96]
Reference	1.77	0	0.41			
Non-clinical diagnosis				2	0.68 [0.22–1.00]	0.89 [0.67–1.00]
Clinical diagnosis				9	0.92 [0.84–1.00]	0.88 [0.78–0.98]
Phase	1.43	0	0.49			
Acute				1	0.69 [0.00–1.00]	0.82 [0.38–1.00]
Chronic				7	0.84 [0.67–1.00]	0.88 [0.76–1.00]
Not specified				3	/	/

Analyses for male proportion and reference standard were not performed because of missing values and limited sample size, respectively.

## 4 Discussion

In recent conflicts, the use of improvised explosive devices and high-energy ammunition, along with enhanced body armor, has shifted clinical focus to blast-related neurological trauma. The damage from the blast wave is primary bTBI (47). Two mechanisms for primary brain injury are proposed. One hypothesis is that shock waves traverse brain tissue, causing acceleration and deformation, and the degree of damage depends on the BSW’s shape, peak overpressure, pulse duration, and tissues’ natural resonant frequencies (48). Another supposition is that shock waves impact the torso, transfer kinetic energy to hydraulic energy in the cardiovascular system, displace blood from the high-pressure body cavity to the low-pressure cranial cavity, and damage cerebral blood vessels and the blood–brain barrier. Owing to these complex pathological changes, acute bTBI symptoms are often easily neglected, increasing the risk of persistent neurological complication. Thus, early diagnosis and targeted intervention are essential to reduce preventable injuries and long-term morbidity in blast-induced trauma survivors (49). Rescue personnel should possess a systematic understanding of the identification and diagnosis of bTBI (3). This meta-analysis demonstrated high diagnostic accuracy for primary bTBI. The pooled sensitivity, specificity and AUC were 0.93 [95%CI: 0.81–0.97], 0.90 [95%CI: 0.80–0.95], and 0.96 [95%CI: 0.94–0.98], respectively. These findings are superior to those of a prior meta-analysis assessing S100B for TBI, which did not specify blast as the injury mechanism, reporting that pooled sensitivity was 89% [95%CI: 83–92%], and the pooled specificity was 32% [95%CI: 26–39%] (50). Among these approaches, laboratory testing methods show excellent diagnostic performance in acute bTBI, however, they still rely on brain tissue or blood samples, which are inaccessible in acute, resource-constrained battlefield scenarios. The same deficiency applies in neuroimaging approaches. Regional resting-state MEG (rs-MEG) slow-wave markers achieved the highest accuracy, demonstrating high sensitivity (>85%) in individually distinguishing chronic and sub-acute mTBI patients with persistent post-concussive syndrome (PCS) from neurologically intact individuals, yet it rely on large, non-portable equipment, which limits its utility on the battlefield (51). VOMS allows a rapid on-site screening, with mVOMS executable within 2 min, but its diagnostic accuracy is relatively lower compared with other methods (52). Among assessment scales, The updated Boston Assessment of TBI Lifetime, Second Edition (BATL-2) performed best. It integrates diagnosis framework from the American Congress of Rehabilitation Medicine (ACRM) and National Institute of Neurological Disorders and Stroke (NINDS), while incorporating modules for military occupational blast exposure and repetitive head impacts (53). In conclusion, scenario-specific selection is recommended over a universal one-size-fits-all approach for practical clinical utility.

Considerable heterogeneity was observed in our study. Subgroup analysis showed that heterogeneity mainly came from human studies, which might be attributed to the strictly controlled experimental conditions and homogeneous samples. Results of the univariate meta-regression suggested that diagnostic modality type contributed moderately to between-study heterogeneity (*p* = 0.05). with visual and olfactory sensory test showing higher specificity than standardized scale-based assessments. Although the mean age were not statistically significant, sample selection bias should be noted, our findings are only applicable to adult U.S. military service members. For animal models, we failed to collect studies using large mammals, such as pigs and goats, to conduct experiments, which is consistent with a recent review (44). Future studies should consider several key confounders, including: race, gender (24), military occupational specialty (MOS) (53), the mechanism of the blast (e.g., type of explosive; distance; evidence of secondary, tertiary, and quaternary injury), the patient’s symptoms (17).

We acknowledge several limitations that may affect the comprehensiveness of our findings. First, only a limited number of biomarker studies provided complete 2×2 contingency data. Second, high heterogeneity was observed, which may reduce transferability. Potential sources of heterogeneity may stem from variation in study design, participant characteristics and outcome assessment approaches. Further studies are warranted to mitigate these differences. Third, most studies enrolled young adult male U.S. military personnel, which restricts extrapolation to civilians, females, or other age groups. Fourth, most included human studies applied retrospective designs, and focused on chronic-stage bTBI cases, while evidence on acute bTBI diagnosis remains scarce. This probably stems from practical challenges in recruiting acute primary bTBI patients under urgent conditions, as the symptoms are easily missed. Fifth, owing to a lack of universal diagnostic reference standard for primary bTBI, we applied a flexible reference criteria to incorporate eligible studies, which inevitably added to the overall between-study heterogeneity. Thus, the results should be interpreted with caution, further studies should aim to address these limitations by refining search methodologies and expanding the scope of included research materials.

Although great progress has been achieved over the past decades, studies focused on primary bTBI remain scarce, and the mechanism is poorly understood (1). Current approaches largely focus on optimization of well-established methods, such as BATL-2, VCU rCDI and VOMS. However, innovative diagnostic measures, especially rapid, non-invasive diagnostic techniques suitable for primary bTBI, are limited. This gap hinders the clinical application of bTBI diagnosis, particularly in emergency settings such as the battlefield, where timely and non-invasive detection is critical. Another finding is the imbalance in research investment across countries. The United States has maintained substantial investment in TBI research, with sustained funding allocated to support in-depth investigations into bTBI, which explains why most studies included in this meta-analysis originated from the U.S. Since 2015, more than $2.1 billion in federal and private funds have been invested in service-connected TBI research across the continuum of care in the USA (44). Whereas, little research about the diagnostic tools development of primary bTBI was found in countries or regions affected by wars and armed conflicts where the incidence of bTBI among both military personnel and civilians is presumably much higher due to repeated blast exposures. Thus, there is an urgent need to develop and validate truly rapid, non-invasive diagnostic modalities for primary bTBI that are suitable for on-site application in the battlefield. As drone attacks become increasingly common in modern warfare, the risk of bTBI among SMs and civilian population in conflict zones continues to rise. Research into rapid diagnosis, early intervention, and preventive strategies of bTBI has therefore become an imperative priority for the global medical community (54). More effort should be devoted to providing better scenario-specific diagnostic measures for the early identification and intervention of bTBI, ultimately reducing the short-term and long-term morbidity of blast trauma survivors worldwide.

## 5 Conclusion

Our findings indicate that current diagnostic methods for primary bTBI are highly accurate. Each method has its own strengths and limitations, meaning their applicability varies across different clinical contexts. Moving forward, greater effort should be put into developing rapid, non-invasive, and precise diagnostic tools for primary bTBI, particularly for use in emergency situations.

## Data Availability

The original contributions presented in the study are included in the article/Supplementary material, further inquiries can be directed to the corresponding authors.

## References

[1] Zasler N. Brain injury: applications from war and terrorism. Brain Inj. (2015) 29:125. doi: 10.3109/02699052.2014.936910

[2] Terrio H Brenner LA Ivins BJ Cho JM Helmick K Schwab K . Traumatic brain injury screening: preliminary findings in a US Army brigade combat team. J Head Trauma Rehabil. (2009) 24:14–23. doi: 10.1097/HTR.0b013e31819581d8, 19158592

[3] Bukowski J Nowadly CD Schauer SG Koyfman A Long B. High risk and low prevalence diseases: blast injuries. Am J Emerg Med. (2023) 70:46–56. doi: 10.1016/j.ajem.2023.05.003, 37207597

[4] David XC Sara IC Henry LL Michael J Barbara JS. The history and evolution of traumatic brain injury rehabilitation in military service members and veterans. Am J Phys Med Rehabil. (2010) 89:688–94. doi: 10.1097/PHM.0b013e3181e722ad, 20647782

[5] Scott SG Belanger HG Vanderploeg RD Massengale J Scholten J. Mechanism-of-injury approach to evaluating patients with blast-related polytrauma. J Am Osteopath Assoc. (2006) 106:265–70. doi: 10.7556/jaoa.2006.106.5.265 16717367

[6] Belding JN Englert RM Fitzmaurice S Jackson JR Koenig HG Hunter MA . Potential health and performance effects of high-level and low-level blast: a scoping review of two decades of research. Front Neurol. (2021) 12:628782. doi: 10.3389/fneur.2021.628782, 33776888 PMC7987950

[7] Farmer CM Krull H Concannon TW Simmons M Pillemer F Ruder T . Understanding treatment of mild traumatic brain injury in the military health system. Rand Health Q. (2017) 6:11. 28845349 10.7249/rhq-06-02-11 PMC5568165

[8] Broglio SP Ferrara MS Sopiarz K Kelly MS. Reliable change of the sensory organization test. Clin J Sport Med. (2008) 18:148–54. doi: 10.1097/JSM.0b013e318164f42a, 18332690

[9] Hoffer ME Gottshall KR Moore R Balough BJ Wester D. Characterizing and treating dizziness after mild head trauma. Otol. Neurotol. (2004) 25:135–8. doi: 10.1097/00129492-200403000-00009, 15021772

[10] Kong L-Z Zhang R-L Hu S-H Lai J-B. Military traumatic brain injury: a challenge straddling neurology and psychiatry. Military medical. Research. (2022) 9:2. doi: 10.1186/s40779-021-00363-y, 34991734 PMC8740337

[11] Mac Donald CL Johnson AM Wierzechowski L Kassner E Stewart T Nelson EC . Outcome trends after US military concussive traumatic brain injury. J Neurotrauma. (2017) 34:2206–19. doi: 10.1089/neu.2016.4434, 27198861 PMC5510713

[12] Schneiderman AI Braver ER Kang HK. Understanding sequelae of injury mechanisms and mild traumatic brain injury incurred during the conflicts in Iraq and Afghanistan: persistent postconcussive symptoms and posttraumatic stress disorder. Am J Epidemiol. (2008) 167:1446–52. doi: 10.1093/aje/kwn068, 18424429

[13] Hoffer ME Balaban C Gottshall K Balough BJ Maddox MR Penta JR. Blast exposure: vestibular consequences and associated characteristics. Otol Neurotol. (2010) 31:232–6. doi: 10.1097/MAO.0b013e3181c993c3, 20009782

[14] Hernandez A Tan C Plattner F Logsdon AF Pozo K Yousuf MA . Exposure to mild blast forces induces neuropathological effects, neurophysiological deficits and biochemical changes. Molecular. Brain. (2018) 11:11. doi: 10.1186/s13041-018-0408-1, 30409147 PMC6225689

[15] Marion DW Curley KC Schwab K Hicks RR. M TBIDW. Proceedings of the military mTBI diagnostics workshop, St. Pete Beach, august 2010. J Neurotrauma. (2011) 28:517–26. doi: 10.1089/neu.2010.1638, 21265587

[16] Fortier CB Amick MM Grande L McGlynn S Kenna A Morra L . The Boston assessment of traumatic brain injury-lifetime (BAT-L) Semistructured interview: evidence of research utility and validity. J Head Trauma Rehabil. (2014) 29:89–98. doi: 10.1097/HTR.0b013e3182865859, 23535389 PMC3997066

[17] Corrigan JD Bogner J. Initial reliability and validity of the Ohio State University TBI identification method. J Head Trauma Rehabil. (2007) 22:318–29. doi: 10.1097/01.HTR.0000300227.67748.77, 18025964

[18] Walker WC Cifu DX Hudak AM Goldberg G Kunz RD Sima AP. Structured interview for mild traumatic brain injury after military blast: inter-rater agreement and development of diagnostic algorithm. J Neurotrauma. (2015) 32:464–73. doi: 10.1089/neu.2014.3433, 25264909

[19] DeCuypere M Klimo P. Spectrum of traumatic brain injury from mild to severe. Surg Clin N Am. (2012) 92:939–57. doi: 10.1016/j.suc.2012.04.005, 22850156

[20] Rowland JA Martindale SL Spengler KM Shura RD Taber KH. Sequelae of blast events in Iraq and Afghanistan war veterans using the Salisbury blast interview: a CENC study. Brain Inj. (2020) 34:642–52. doi: 10.1080/02699052.2020.1729418, 32096666 PMC9007162

[21] Haran FJ Slaboda JC King LA Wright WG Houlihan D Norris JN. Sensitivity of the balance error scoring system and the sensory organization test in the combat environment. J Neurotrauma. (2016) 33:705–11. doi: 10.1089/neu.2015.4060, 26560740

[22] Mucha A Collins MW Elbin RJ Furman JM Troutman-Enseki C DeWolf RM . A brief vestibular/ocular motor screening (VOMS) assessment to evaluate concussions preliminary findings. Am J Sports Med. (2014) 42:2479–86. doi: 10.1177/0363546514543775, 25106780 PMC4209316

[23] Kontos AP Zynda AJ Minerbi A. Comparison of vestibular/ocular motor screening (VOMS) and computerized eye-tracking to identify exposure to repetitive head impacts. Mil Med. (2024) 189:2291–7. doi: 10.1093/milmed/usae065, 38531077

[24] Maxin AJ Lim DH Kush S Carpenter J Shaibani R Gulek BG . Smartphone Pupillometry and machine learning for detection of acute mild traumatic brain injury: cohort study. JMIR Neurotechnol. (2024) 3:e58398. doi: 10.2196/58398, 41341245 PMC12671303

[25] Maxin AJ Gulek BG Lee CE Lim D Mariakakis A Levitt MR . Validation of a smartphone Pupillometry application in diagnosing severe traumatic brain injury. J Neurotrauma. (2023) 40:2118–25. doi: 10.1089/neu.2022.0516, 37464770

[26] Kim JJ Gean AD. Imaging for the diagnosis and Management of Traumatic Brain Injury. Neurotherapeutics. (2011) 8:39–53. doi: 10.1007/s13311-010-0003-3, 21274684 PMC3026928

[27] Isokuortti H Iverson GL Silverberg ND Kataja A Brander A Öhman J . Characterizing the type and location of intracranial abnormalities in mild traumatic brain injury. J Neurosurg. (2018) 129:1588–97. doi: 10.3171/2017.7.JNS17615, 29328003

[28] Asken BM DeKosky ST Clugston JR Jaffee MS Bauer RM. Diffusion tensor imaging (DTI) findings in adult civilian, military, and sport-related mild traumatic brain injury (mTBI): a systematic critical review. Brain Imaging Behav. (2018) 12:585–612. doi: 10.1007/s11682-017-9708-9, 28337734

[29] Hannawi Y Stevens RD. Mapping the connectome following traumatic brain injury. Curr Neurol Neurosci Rep. (2016) 16:44. doi: 10.1007/s11910-016-0642-9, 27021773

[30] Shenton ME Hamoda HM Schneiderman JS Bouix S Pasternak O Rathi Y . A review of magnetic resonance imaging and diffusion tensor imaging findings in mild traumatic brain injury. Brain Imaging Behav. (2012) 6:137–92. doi: 10.1007/s11682-012-9156-5, 22438191 PMC3803157

[31] Mac Donald CL Johnson AM Cooper D Nelson EC Werner NJ Shimony JS . Detection of blast-related traumatic brain injury in U.S. military personnel. N Engl J Med. (2011) 364:2091–100. doi: 10.1056/NEJMoa1008069, 21631321 PMC3146351

[32] Vascak M Jin XT Jacobs KM Povlishock JT. Mild traumatic brain injury induces structural and functional disconnection of local neocortical inhibitory networks via Parvalbumin interneuron diffuse axonal injury. Cereb Cortex. (2018) 28:1625–44. doi: 10.1093/cercor/bhx058, 28334184 PMC5907353

[33] Pham N Sawyer TW Wang YS Jazii FR Vair C Taghibiglou C. Primary blast-induced traumatic brain injury in rats leads to increased prion protein in plasma: a potential biomarker for blast-induced traumatic brain injury. J Neurotrauma. (2015) 32:58–65. doi: 10.1089/neu.2014.3471, 25058115 PMC4273182

[34] Shi QX Chen B Nie C Zhao ZP Zhang JH Si SY . A novel model of blast induced traumatic brain injury caused by compressed gas produced sustained cognitive deficits in rats: involvement of phosphorylation of tau at the Thr205 epitope. Brain Res Bull. (2020) 157:149–61. doi: 10.1016/j.brainresbull.2020.02.002, 32044361

[35] Samson K. FDA approves first blood test for brain bleeds after mild TBI/concussion. Neurol Today. (2018) 18:12–8. doi: 10.1097/01.NT.0000532091.01255.0b

[36] Tschiffely AE Statz JK Edwards KA Goforth C Ahlers ST Carr WS . Assessing a blast-related biomarker in an operational community: glial fibrillary acidic protein in experienced Breachers. J Neurotrauma. (2020) 37:1091–6. doi: 10.1089/neu.2019.6512, 31642374 PMC7364308

[37] Agoston DV Elsayed M. Serum-based protein biomarkers in blast-induced traumatic brain injury spectrum disorder. Front Neurol. (2012) 3:107, . 22783223 10.3389/fneur.2012.00107 PMC3390892

[38] Kobeissy F Mondello S Tümer N Toklu HZ Whidden MA Kirichenko N . Assessing neuro-systemic & behavioral components in the pathophysiology of blast-related brain injury. Front Neurol. (2013) 4:186. doi: 10.3389/fneur.2013.00186, 24312074 PMC3836009

[39] Arun P Abu-Taleb R Oguntayo S Tanaka M Wang Y Valiyaveettil M . Distinct patterns of expression of traumatic brain injury biomarkers after blast exposure: role of compromised cell membrane integrity. Neurosci Lett. (2013) 552:87–91. doi: 10.1016/j.neulet.2013.07.047, 23933206

[40] Oris C Kahouadji S Durif J Bouvier D Sapin V. S100B, actor and biomarker of mild traumatic brain injury. Int J Mol Sci. (2023) 24:6602. doi: 10.3390/ijms24076602, 37047574 PMC10095287

[41] Bazarian JJ Blyth BJ He H Mookerjee S Jones C Kiechle K . Classification accuracy of serum Apo A-I and S100B for the diagnosis of mild traumatic brain injury and prediction of abnormal initial head computed tomography scan. J Neurotrauma. (2013) 30:1747–54. doi: 10.1089/neu.2013.2853, 23758329 PMC4047844

[42] U.S. Food and Drug Administration, Agency for Healthcare Research and Quality. Using the PICOTS Framework to Strengthen Evidence Gathered in Clinical Trials—Guidance from the AHRQ’s Evidence-based Practice Centers. Available online at: https://www.fda.gov/media/109448/download. (Accessed June 08, 2026).

[43] Arya S Kaji AH Boermeester MA. PRISMA reporting guidelines for Meta-analyses and systematic reviews. JAMA Surg. (2021) 156:789–90. doi: 10.1001/jamasurg.2021.0546, 33825806

[44] Hoch E Martinez J Bakhshi R Hieber M Presser A Tartaglia T. A review of U.S. military traumatic brain injury studies: trends, gaps, and opportunities. Santa Monica, CA: RAND Corporation (2025) Available online at: https://www.rand.org/pubs/research_reports/RRA4199-1.html 10.7249/rhq-13-02-07 PMC12978667 41821668

[45] National Academies of Sciences, Engineering, and Medicine. Improving Diagnosis in Health Care. Washington, DC: The National Academies Press (2015).

[46] Whiting PF Rutjes AWS Westwood ME Mallett S Deeks JJ Reitsma JB . QUADAS-2: a revised tool for the quality assessment of diagnostic accuracy studies. Ann Intern Med. (2011) 155:529–36. doi: 10.7326/0003-4819-155-8-201110180-00009, 22007046

[47] START. Global Terrorism Database 1970–2020 (2022).

[48] French LM Taber KH Helmick K Hurley RA Warden DL. "Traumatic brain injury in the military population". In: Combat and Operational Behavioral Health. Fort Sam Houston, TX, USA: Department of the Army The Borden Institute (2015)

[49] Shetty AK Mishra V Kodali M Hattiangady B. Blood brain barrier dysfunction and delayed neurological deficits in mild traumatic brain injury induced by blast shock waves. Front Cell Neurosci. (2014) 8:232. doi: 10.3389/fncel.2014.00232, 25165433 PMC4131244

[50] Karamian A Farzaneh H Khoshnoodi M Maleki N Karamian A Stufflebeam S . Diagnostic accuracy of S100B in predicting intracranial abnormalities on CT imaging following mild traumatic brain injury: a systematic review and Meta-analysis. Neurocrit Care. (2025) 42:1025–42. doi: 10.1007/s12028-024-02189-7, 39776345

[51] Huang MX Huang CW Harrington DL Robb-Swan A Angeles-Quinto A Nichols S . Resting-state magnetoencephalography source magnitude imaging with deep-learning neural network for classification of symptomatic combat-related mild traumatic brain injury. Hum Brain Mapp. (2021) 42:1987–2004. doi: 10.1002/hbm.25340, 33449442 PMC8046098

[52] Kranfli AA Cerda C Moore C Knicely K Kontos AP Zynda AJ . Using the modified vestibular/ocular motor screening tool to identify blast exposure effects in military service members. Mil Med. (2025) 191:e1131–9. doi: 10.1093/milmed/usaf544, 41206909

[53] Colaizzi T Kenna A Knight A Clermont C Currao A Fortier CB. The Boston assessment of traumatic brain injury lifetime, second edition (BATL-2): development and initial psychometric evaluation in Post-9/11 military veterans. J Head Trauma Rehabil. (2025) 41:235–45. doi: 10.1097/HTR.0000000000001112, 41051978 PMC13143359

[54] Owens BD Kragh JF Jr Wenke JC Macaitis J Wade CE Holcomb JB. Combat wounds in operation Iraqi freedom and operation enduring freedom. J Trauma. (2008) 64:295–9. doi: 10.1097/TA.0b013e318163b875, 18301189

[55] Diociasi A Iaccarino MA Sorg S Lubin EJ Wisialowski C Dua A . Distinct functional MRI connectivity patterns and cortical volume variations associated with repetitive blast exposure in special operations forces members. Radiology. (2025) 315:e233264. doi: 10.1148/radiol.233264, 40167438

[56] Iverson GL Ivins BJ Karr JE Crane PK Lange RT Cole WR . Comparing composite scores for the ANAM4 TBI-MIL for research in mild traumatic brain injury. Arch Clin Neuropsychol. (2020) 35:56–69. doi: 10.1093/arclin/acz021, 31063188

[57] Capó-Aponte JE Tarbett AK Urosevich TG Temme LA Sanghera NK Kalich ME. Effectiveness of computerized oculomotor vision screening in a military population: pilot study. J Rehabil Res Dev. (2012) 49:1377–98. doi: 10.1682/JRRD.2011.07.0128, 23408219

[58] Xydakis MS Mulligan LP Smith AB Olsen CH Lyon DM Belluscio L. Olfactory impairment and traumatic brain injury in blast-injured combat troops a cohort study. Neurology. (2015) 84:1559–67. doi: 10.1212/WNL.0000000000001475, 25788559 PMC4408285

[59] Martindale SL Belding JN Crawford CD Rowland JA. Validation of military occupational specialty as a proxy for blast exposure using the Salisbury blast interview. J Neurotrauma. (2023) 40:2321–9. doi: 10.1089/neu.2023.0067, 37058360

[60] Ge ML Wang YY Wu T Li HB Yang CY Chen TA . Serum-based Raman spectroscopic diagnosis of blast-induced brain injury in a rat model. Biomed Opt Express. (2023) 14:3622–34. doi: 10.1364/BOE.495285, 37497497 PMC10368048

[61] Ge ML Wang YY Wu T Li HB Yang CY Wang ZL . Raman spectroscopic diagnosis of blast-induced traumatic brain injury in rats combined with machine learning. Spectrochimica Acta part a-molecular and biomolecular. Spectroscopy. (2024) 304:123419. doi: 10.1016/j.saa.2023.123419, 37738762

[62] Wang YY Wang GQ Xu DG Jiang BZ Ge ML Wu LM . Terahertz spectroscopic diagnosis of early blast-induced traumatic brain injury in rats. Biomed Opt Express. (2020) 11:4085–98. doi: 10.1364/BOE.395432, 32923030 PMC7449730

