# Supplementary material for: Diagnostic accuracy of blast-induced traumatic brain injury: a systematic review and meta-analysis
Source: Front Neurol. 2026 Jul 13;17:1845916. doi: 10.3389/fneur.2026.1845916 (PMC13402129; doi:10.3389/fneur.2026.1845916)
Supplement: Supplementary file 1 [file Supplementary_file_1.DOCX]

Supplementary Material

# Supplementary Figures and Tables

## Supplementary Table

## Supplementary Table1. Search strategy of bTBI diagnostic methods.

| **Database** | **Search strategy** | **Result** |
| --- | --- | --- |
| Pubmed | #1 ((AUC[Title/Abstract]) OR (sensi*[Title/Abstract]) OR (specif*[Title/Abstract])); #2 (((blast[Title/Abstract]) OR (explosi*[Title/Abstract])) OR (shock wave[Title/Abstract]));#3 ((((TBI[Title/Abstract]) OR (brain injury[Title/Abstract])) OR (concussion[Title/Abstract])) OR (trauma* brain injury[Title/Abstract])); #4 #1 AND #2 AND #3; #5 (severit*[Title/Abstract] OR diagnosis[Title/Abstract] OR Detect*[Title/Abstract] OR Screen*[Title/Abstract] OR Assess*[Title/Abstract] OR Identify*[Title/Abstract] OR Evaluate*[Title/Abstract]); #6 #4 AND #5; #7 ("case report"[Publication Type] OR "systematic review"[Publication Type] OR "meta analysis"[Publication Type])；#8 #6 NOT #7 | 293 |
| Web of Science | 1: TS=("AUC" OR "sensi*" OR "specif*")Date Run: Thu Jan 15 2026 15:31:27 GMT+0800            Results: 8089887；2: TS=("blast" OR "explosi*" OR "shock wave")Date Run: Thu Jan 15 2026 15:31:35 GMT+080Results: 213226；3: TS=("TBI" OR "brain injury" OR "concussion" OR "trauma* brain injury")Date Run: Thu Jan 15 2026 15:31:45 GMT+0800  Results: 144297；4: TS=("severit*" OR "diagnosis" OR "detect*" OR "screen*" OR "assess*" OR "identify*" OR "evaluate*")Date Run: Thu Jan 15 2026 15:31:53 GMT+0800 Results: 15581032；5: #1 AND #2 AND #3 AND #4Date Run: Thu Jan 15 2026 15:31:59 GMT+0800 Results: 343 | 343 |
| Defense Technical Information Center | (TI=(AUC OR sensi* OR specif*)ORAB=(AUC OR sensi* OR specif*))AND(TI=(blast OR explosi*OR"shock wave") OR AB= (blast OR explosi* OR "shock wave")) AND (TI=( TBI OR"brain injury" OR concussion OR "trauma* brain injury") OR AB=(TBI OR "brain injury" OR concussion OR "trauma* brain injury")) AND ( TI=( severit* OR diagnosis OR Detect* OR Screen* OR Assess* OR Identify* OR Evaluate*) OR AB=( severit* OR diagnosis OR Detect* OR Screen* OR Assess* OR Identify* OR Evaluate*)) NOT PT=( "case report" OR "systematic review" OR "meta analysis") | 405 |
| Cochrance library | (AUC OR sensi* OR specif*):ti,ab,kw AND (blast OR explosi* OR "shock wave"):ti,ab,kw AND (TBI OR "brain injury" OR concussion OR "trauma* brain injury"):ti,ab,kw AND (severit* OR diagnosis OR detect* OR screen* OR assess* OR identify* OR evaluate*):ti,ab,kw (Word variations have been searched) | 32 |

## Supplementary Figures


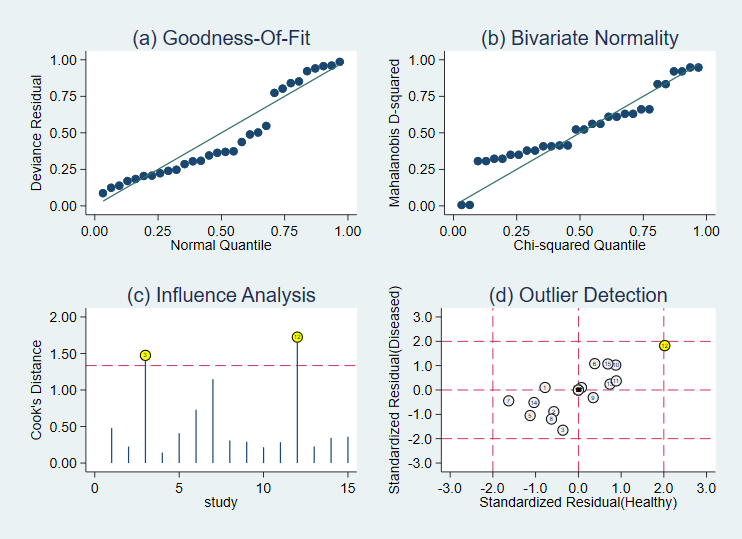


**Supplementary Figure 1.** Diagnostic Model Validation and Residual Analysis.This figure validates the diagnostic model through four analytical perspectives. Panel (a) (Goodness-Of-Fit) shows a quantile-quantile plot of deviance residuals against normal quantiles, indicating good residual normality. Panel (b) (Bivariate Normality) plots Mahalanobis D-squared against chi-squared quantiles, confirming bivariate normality of the dataset. Panel (c) (Influence Analysis) uses Cook's Distance to identify influential studies (peaked at ~10), while Panel (d) (Outlier Detection) highlights potential outliers via standardized residuals for diseased/healthy groups, ensuring model robustness.


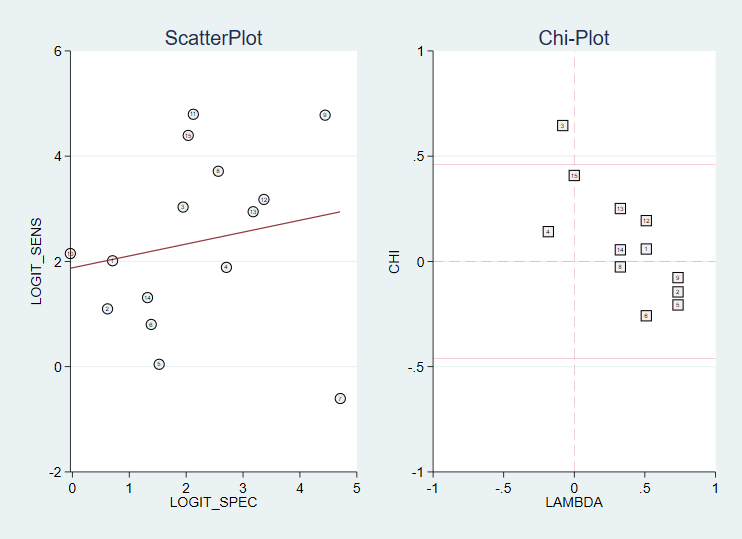


**Supplementary Figure 2.** Bivariate analysis of diagnostic test performance and heterogeneity. The left picture is a scatter plot of log sensitivity and log specificity. the red regression line shows the overall trend between these two metrics. The right picture shows the heterogeneity statistic, chi value versus the effect size. Dashed red lines at LAMBDA=0 and CHI=0 serve as references to identify outliers and assess study-level variability.


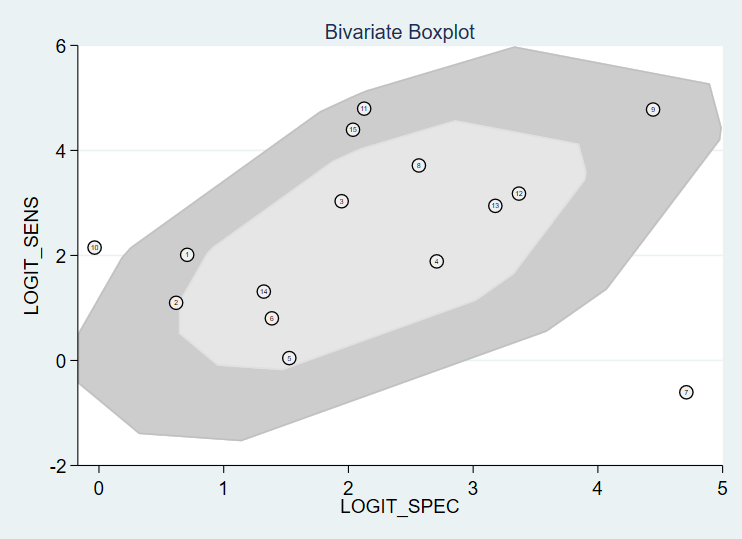


**Supplementary Figure 3.** Bivariate boxplot of Diagnostic Test Performance. This bivariate boxplot illustrates the joint distribution of logit-transformed sensitivity (LOGIT_SENS, y-axis) and specificity (LOGIT_SPEC, x-axis) across diagnostic studies. Numbered points represent individual study estimates, while the nested shaded regions denote concentric quantile layers: the inner light-gray area marks the central data cluster, and the outer dark-gray area reflects the full distribution, visualizing the overall spread and central tendency of diagnostic performance.
